# Dance/movement therapy as a holistic approach to diminish health discrepancies and promote wellness for people with schizophrenia: a review of the literature

**DOI:** 10.12688/f1000research.127377.2

**Published:** 2023-08-01

**Authors:** Jacelyn Biondo

**Affiliations:** 1 Family and Community Medicine, Thomas Jefferson University, Philadelphia, PA, 19107, USA

**Keywords:** dance/movement therapy, schizophrenia, mind-body connection, interoception, healthcare disparities, embodiment, neurobiology

## Abstract

Individuals with a diagnosis of schizophrenia face a myriad of obstacles to wellness, beginning with diagnostic discrepancies including over- and misdiagnoses on the schizophrenia spectrum. People with schizophrenia experience profound amounts of stigmatization from the general population, their healthcare providers, and even themselves. Such stigmatization creates a barrier for wellness, poorer prognoses, and often limits adherence to physical and mental healthcare. Moreover, it can exacerbate the already stifling symptomatology of their diagnoses, including specific bodily-related symptomatology. Oftentimes, a diagnosis of schizophrenia disrupts one’s relationship with their body including a diminished mind-body connection, decreased interoceptive awareness, and thus unsuccessful intra- and interpersonal relationships. Some recent research suggests the use of mind-body therapies, however, if these practices are internalizing, they may not be appropriate for people with schizophrenia experiencing more acute symptomatology excluding them from treatment. Dance/movement therapy (DMT) is an embodied psychotherapeutic treatment option that can support participants in improving mind-body connection, social relationships, and self-regulatory skill development. Research on DMT has shown promising results for people with schizophrenia, however such research is limited and would benefit from increased studies that particularly measure the effects of DMT on mind-body connection and increased interoception for people with schizophrenia. Moreover, integrative and collaborative treatment models that couple DMT and biofeedback may further our understanding of the physiological and neurological effects of DMT interventions for people with schizophrenia and beyond. This review will examine the recent literature on health inequities for people with schizophrenia, their specific body-based disruptions and needs, and DMT as a promising treatment model, particularly when coupled with biofeedback.

## Introduction

Individuals diagnosed with schizophrenia face discrepancies regarding health care services at a rate much higher than the general population ( Moore *et al.*, 2015; Oud & Jong, 2017; Tan *et al*., 2021). The inequities they face include both physical ( Moore *et al.*, 2015; Sølvhøj *et al*., 2021) and mental health care ( Alang, 2019; Ivanova, 2021; Metzl & Roberts, 2014; Rössler, 2016), and contribute to a decrease in general wellbeing and thus quality of life. People with schizophrenia have specific and additional needs due to their complicated symptomatology inclusive of body-based dysregulations ( Biondo *et al*., 2021; Davis, 2019; Klaver & Dijkerman, 2016). Dance/movement therapy (DMT) is a strengths- and body-based psychotherapeutic approach that uses dance, movement, and the body to support mind-body connection, emotional processing, and holistic wellness ( Levy, 1988). The formal profession of DMT began in the early 1940s during which Marian Chace worked at St. Elizabeth’s Hospital in Washington D.C. Here, Chace provided dance therapy sessions for individuals diagnosed with ’shell shock’ returning from World War II and those diagnosed with schizophrenia prior to the use of psychotropic medication ( American Dance Therapy Association, 2021). Since DMT incorporates both verbal and non-verbal forms of communication in its processes, it can be an inclusive practice for individuals with schizophrenia who may, due to their symptomatology, have styles of communication that differ from the societal norm ( Biondo *et al*., 2021). Joining through movement facilitates increased attunement, which is a necessary component to foster self-regulation and encourage healthy interpersonal skills. Additionally, DMT provides the framework to access embodiment and neurophysiological theories, which supports the multifaceted needs of individuals diagnosed with schizophrenia. One of the primary tenets of DMT is that the mind and body are interrelated and thus have a relationship that inform wellness. DMT is an active and embodied treatment option to increase mind-body connection. Dance/movement therapists have foundational knowledge rooted in embodied, trauma-informed, nonverbal psychotherapeutic practices ( Homann, 2020; Koch, 2017). This paper will review the current literature on physical and mental health care inequities for people with schizophrenia, bodily disturbances associated with the diagnosis, and ways in which DMT, possibly coupled with biofeedback, can be a holistic approach to treatment.

## Discussion

### Mental health disparities

Mental health disparities for people begin with diagnostics. This is largely due to the structural racism that occurs within mental health facilities. Systemically racist underpinnings of mental health care facilities cause undue stress for People of Color (POC), particularly Black and African American men ( Alang, 2019; Asonye *et al*., 2020; Harnett & Ressler, 2021; Metzl & Roberts, 2014; Misra *et al.,* 2022). Over- and misdiagnosis of African Americans, particularly as it pertains to schizophrenia, have been consistent even with the adjustments made to the Diagnostic and Statistical Manual (DSM) from its inception in 1952 and continuing through each iteration. Pre-Civil Rights era, a diagnosis of schizophrenia was largely associated with middle-class, white housewives experiencing symptoms of regressive behaviors, disrupted moods, and an inability to care for the home ( Asonye *et al*., 2020; Metzl & Roberts, 2014). However, this shifted dramatically in the mid-1950s in parallel with the Civil Rights movement. At that point in time, schizophrenia became a diagnosis largely relegated to Black and African American men at rates of 65% more frequently than white men. By the 1970s and 1980s, with the release of the DSM II and III consecutively, Black and African American men were five to seven times more likely to receive a diagnosis of paranoid schizophrenia than their white male counterparts ( Asonye *et al.,* 2020; Metzl & Roberts, 2014). Racial discrepancies continued through changes in diagnostic criteria of the DSM IV, with poorly identified rationale for the racial differences in diagnostics ( Strakowski *et al*., 1996). Furthermore, Hampton (2007) noted that a diagnosis of schizophrenia of African American individuals over white individuals increased due in part to the narrowing of criteria. Both historically and presently, Black men are more likely to receive a diagnosis of schizophrenia than white men with the same presenting symptoms. There is an over–diagnosis of schizophrenia and an under–diagnosis of mood disorders for Black and African American men ( Asonye *et al.,* 2020; Metzl & Roberts, 2014). This is partially attributed to the mistrust and stigmatization psychiatrists and other mental health care workers have towards POC and people with severe mental illnesses ( Alang, 2019; Asonye *et al.,* 2020; Harnett & Ressler, 2021; Metzl & Roberts, 2014; Misra *et al.,* 2022).

Individuals with schizophrenia experience stigmatization at rates higher than other individuals with mental health diagnoses ( Mannarini *et al.,* 2022; Rössler, 2016; Valery & Prouteau, 2020). Ivanova (2021) defined stigmatization as “a mark of shame, disgrace or disapproval which results in an individual being rejected, discriminated against, and excluded from participating in a number of different areas of society” (p. 47). Historically, people who experienced mental illness were treated extremely poorly with consequences of being held against their will, tortured, or even killed ( Rössler, 2016). Although these practices have ceased, the fear of people with mental illness, particularly those with a diagnosis of schizophrenia, remains. The association of violence or aggression with individuals with schizophrenia is incorrect ( Ivanova, 2021; Mannarini *et al.,* 2022; Rössler, 2016). Research shows that people with schizophrenia are 75-120% more likely to be a victim of violence than the general population ( Ivanova, 2021).

Stigmatizating behaviors extend to psychiatrists, making them less likely to meet in a clinical context with individuals diagnosed with schizophrenia ( Rössler, 2016). In events where people with schizophrenia have sought mental health services, some reported feeling “devalued, dismissed, and dehumanized by healthcare professionals” ( Ivanova, 2021, p. 49). This speaks to the level of exclusion people with schizophrenia experience, deeply rooted in stigmatization ( Lincoln *et al*., 2021). Individuals with schizophrenia are often excluded from medical decision making and feel coerced by medical care practitioners ( Ivanova, 2021). Both medical decision making and social exclusion can have detrimental effects on mental and physical wellness, leading to an increase in symptomatology ( Lincoln *et al*., 2021). Research has shown a direct relationship between feelings of social exclusion with negative neurobiological responses ( Lincoln *et al*., 2021; Metzl & Roberts, 2014). Beyond medical care, researchers have often excluded individuals with schizophrenia from their studies due to the presence of positive symptomatology or an exacerbation of symptomatology indicating they may be unable to manage the intervention or treatment ( Biondo *et al*., 2021). This prevents people with schizophrenia from informing, testing, and responding to possible treatment protocols that could provide them with more effective care. A less medical and more psychosocial approach to support could mediate some of this stigmatization, and resultant exclusion, exhibited by health care providers ( Ivanova, 2021). Unfortunately, these undesirable behaviors of medical professionals traverse beyond mental health care and into the realm of physical health care as well ( Kohn *et al*., 2022; Oud & Jong, 2017; Sølvhøj *et al*., 2021; Swildens *et al.*, 2016).

### Physical health disparities

Stigmatization affects people with schizophrenia on personal and social levels. In a survey inclusive of 27 countries, people with schizophrenia reported discriminatory behaviors in their personal relationships at a rate of 50% and in job related relationships at a rate of 67% ( Rössler, 2016). This stigmatization traverses into the relationship between people with schizophrenia and their health care providers, who have a higher rate of negative beliefs about people with schizophrenia than the general population ( Mannarini *et al.,* 2022; Rössler, 2016; Valery & Prouteau, 2020). Consistent stigmatizing behaviors from others can lead to self-stigmatization behaviors for individuals with schizophrenia—compounding a diminished sense of self-esteem and self-efficacy ( Mannarini *et al.,* 2022; Rössler, 2016; Valery & Pruteau, 2020). This ultimately inhibits and limits the care that people with schizophrenia are seeking and receiving, and contributes to negative effects on their overall prognosis ( Ivanova, 2021; Mannarini *et al.,* 2020).

People with a diagnosis of schizophrenia have a lifespan 10-20 years shorter than the general population and have a 2.5 times higher risk of death compared to the general population ( Moore *et al*., 2015; Oud & Jong, 2017). The co-morbidity rate for schizophrenia and somatic disorders—including diabetes mellitus, cardiovascular diseases, and respiratory diseases—is the highest level of physical health co-morbidity as compared to other people diagnosed with severe mental illness ( Tan *et al*., 2021). There are many factors that contribute to inequities in health care for people with schizophrenia, including disparities in access, use, and provision of services ( Kohn *et al*., 2022) and general limited use of somatic health care practitioners ( Swildens *et al*., 2016). Somatic diseases are often underdiagnosed and under-treated amongst people with severe mental illness, particularly schizophrenia ( Kohn *et al*., 2022; Laursen, 2019; Oud & Jong, 2017) In addition to the poor medical treatment adherence rate ( Kohn *et al*., 2022; Laursen, 2019), people with schizophrenia also received poorer care, less preventative and curative screening processes, and less prescriptions ( Laursen, 2019).

One of the causes of poorer quality of care is the ongoing stigmatization of people with schizophrenia ( Kohn *et al*., 2022; Oud & Jong, 2017; Sølvhøj *et al*., 2021; Swildens *et al*., 2016). Individuals diagnosed with schizophrenia feel that they are spoken down to or even patronized by doctors when they are seeking treatment. Furthermore, due to differences in communication styles and preferences, people with schizophrenia have reported feeling uncomfortable expressing their concerns to doctors for fear of being physically or emotionally hurt or becoming recipients of other forms of stigmatization ( Kohn *et al*., 2022).

Stigmatizing behaviors compound communication difficulties, reducing the ability for people with schizophrenia to express their physical concerns readily ( Kohn *et al*., 2022). This surge in stress may also result in the emergence of or contribution to somatic diseases ( Oud & Jong, 2017; Vancampfort *et al*., 2017). Oftentimes, such communication difficulties heighten isolative behaviors. Negative symptomatology of schizophrenia, including social withdrawal and lack of spontaneity, can interfere with appropriate physical health care. These symptoms encourage a sedentary lifestyle, which typically decreases one’s ability to report physical health symptoms accurately and readily ( Moore *et al*., 2015; Oud & Jong, 2017; Vancampfort *et al*., 2017). Moreover, a sedentary lifestyle coupled with metabolic effects of antipsychotic medications contribute to people with schizophrenia being overweight, furthering their propensity towards developing a somatic disease ( Laursen, 2019; Moore *et al*., 2015; Oud & Jong, 2017). Both the negative symptomatology and the negative side effects of antipsychotics trigger poor self-esteem ( Vancampfort *et al*., 2017) and a loss of hope for people with schizophrenia ( Oud & Jong, 2017). The added complication of having a limited social support in addition to the aforementioned complications all act as barriers to accessible, quality health care of people with schizophrenia ( Vancampfort *et al*., 2017).

### Body symptomatology

People with schizophrenia often have specific care needs due to movement dysregulations that are associated with their diagnosis ( Biondo *et al*., 2021; Davis, 2019). The Diagnostic and Statistical Manual of Mental Disorders, Fifth edition (DSM-5) notes macro-level movement dysregulations such as holding bizarre postures or grossly disorganized movements ( American Psychological Association [APA], 2013); however, there are many more subtle and disruptive bodily disturbances associated with schizophrenia including movement fragmentation, unsynchronized movement, subtle and bizarre facial expressions, or limited ability for spontaneous movements or gestures ( Biondo *et al*., 2021; Davis, 2019). These movement manifestations can be attributed to either pharmacological side effects or naturally occurring abnormal involuntary movements of the nervous system ( Cruz, 1995/ 2009).

### Bodily disturbances and diminished mind-body connection and interoception

Diminished body awareness often correlates with higher symptomatology, which thus contributes to further bodily disruptions ( Costantini *et al*., 2020). This cycle of symptomatology and body disturbances disallows healthy mind-body connectivity for people with schizophrenia. Typically, bodies and brains communicate with one another in order to maintain a homeostatic relationship within one’s self. This reciprocal communication pathway allows for self-regulatory actions as needed ( Yao & Thakkar, 2022). These and other body disruptions for people with schizophrenia contribute to a significant decrease in mind-body connection, which interfere with wellness on multiple levels.

Without a grounded sense of bodily self, people with schizophrenia often experience a diminished ego or sense of self ( Benson *et al*., 2019; Biondo *et al*., 2021; Costantini *et al*., 2020; Torregrossa *et al.*, 2022; Yao & Thakkar, 2022). Further compounding a limited sense of self for people with schizophrenia is their often disrupted interoceptive awareness. Healthy interoceptive awareness is associated with brain functioning and can be affected by a diagnosis of schizophrenia. Interoception is the internal and physiological awareness of one’s bodily sensations ( Ardizzi *et al*., 2016; Torregrossa *et al*., 2022; Yao & Thakkar, 2022), that supports an integrative processes of internal body cues ( Yao & Thakkar, 2022). Thus, without interoceptive awareness, people with schizophrenia often experience increased positive symptomatology ( Torregrossa *et al.*, 2022). Individuals diagnosed with schizophrenia may experience difficulty with interpreting their own body signals as well as those of others ( Oud & Jong, 2017). This also attributes to the disruption of body boundaries ( Benson *et al.*, 2019; Costantini *et al*., 2020); at times they do not understand where their bodies end and others begin. A diminished sense of boundaries can have both intra- ( Ardizzi *et al*., 2016; Benson *et al.*, 2019; Costantini *et al*., 2020; Torregrossa *et al*., 2022) and inter-personal ( Torregrossa *et al*., 2022) difficulties. On an intrapersonal level, individuals may experience a disruption of their body in shape, size, location, ownership ( Costantini *et al*., 2020), and pain levels ( Kohn *et al*., 2022; Yao & Thakkar, 2022)—they may even have difficulty recognizing their own voice or face ( Benson *et al*., 2019). Inhibited interoception alters one’s ability to appropriately regulate the autonomic nervous system, which is essential to survival. It similarly affects the response rate of receiving external sensory signals—exteroception—and the ability to process cognition and emotion ( Ardizzi *et al*., 2016; Yao & Thakkar, 2022). Ardizzi *et al.* (2016) noted that “interoceptive accuracy … also appears to be involved in the autonomic regulation during social interactions and individual resilience ability,” (p. 2) further compounding interpersonal relationships. In order to fully understand and relieve symptomatology of the body for people with schizophrenia, it is argued we need to use the body as a tool for exploration and healing ( Klaver & Dijkerman, 2016). Yao and Thakkar (2022) noted that “exploring how persons with schizophrenia experience their bodies and interpret their bodily signals is potentially key to understanding illness mechanisms” (p. 758).

### Mind-body treatments

In consideration of attention to both physical and mental health when working with individuals with schizophrenia, researchers have implemented mind-body therapies to mitigate symptoms of schizophrenia ( Behere *et al*., 2019; Sabe *et al*., 2019; Vogel *et al*., 2019). Mind-body treatment interventions have proven effective for people with schizophrenia; however, one caveat to inclusion into these practices is stabilization of acute symptomatology of schizophrenia. Therefore, mind-body connection interventions for people with schizophrenia often focus on the diminishment of negative symptomatology ( Behere *et al*., 2019; Sabe *et al*., 2019; Vogel *et al*., 2019). This is due, in part, to the internalization that accompanies some of the more meditative practices of mind-body connectivity, which may increase positive symptomatology. In their review, Behere *et al*. (2019) suggested that yoga may be effective in improving negative symptomatology, however, there was no mention of whether the interventions had an effect on mind-body connectivity. In their systematic review ( *K* = 15; *N* = 1081), Sabe *et al*. (2019) noted that that mind-body therapies, such as tai chi, yoga, and qigong, support increased self-efficacy and agency, and decrease stress for people experiencing negative symptoms of schizophrenia.

Vogel *et al*. (2019) reviewed mind-body and aerobic exercises in their meta–analysis ( *K* = 22; *N* = 1249), noting that typically psychopharmacological and some psychological interventions are ineffective at treating negative symptomatology of schizophrenia. They further posited that physical exercise can have beneficial effects on neural pathways as these neural pathways relate to the internal reward system. Moreover, physical exercise was shown to be effective at decreasing negative symptoms and feelings of depression, while increasing working memory, social cognition, attention, cardiorespiratory fitness, and improving Positive and Negative Symptom Scale (a medical scale used for measuring symptom severity of patients with schizophrenia) scores ( Vogel *et al*., 2019). The authors concluded that activation is an important component of wellness for people with schizophrenia and that mind-body exercise shows promise as an intervention for this population.

### Biofeedback

Biofeedback is an underutilized therapeutic tool that affirms mind-body connectivity while giving recipients and clinicians in-the-moment health related information. This further substantiates the notion that physical health can directly affect mental and emotional aspects and vice versa ( Austad & Gendron, 2018). Although there is some consideration for the mind-body connection and how it affects wellness, many forms of traditional psychotherapy do not enter into the therapeutic process with the body at the forefront ( Austad & Gendron, 2018; Fiskum, 2019).

As self-regulation is deeply rooted in the body and neurophysiology, biofeedback may be a functional approach to physiological regulation. This can elevate awareness of the mind-body connection while providing adaptive tools for self-regulation. Provision of such regulation tools can further self-awareness and improve physical and mental health ( Austad & Gendron, 2018). Measurements of high Heart Rate Variability (HRV), and signals of lower cardiac complexity, can provide data suggestive of an increased capacity for emotion regulation and overall psychological wellbeing ( Fiskum, 2019). HRV biofeedback may have a positive effect on “synchronized blood-flow oscillations … further strengthening the functional connectivity between brain areas” ( Fiskum, 2019, p. 418).

### Neurofeedback

King and Parada (2020) suggested using mobile brain/body imaging (MoBI) as a more pragmatic way of measuring neuroscientific activation resulting from an intervention within the CATs. Mobile brain/body imaging could be a process through which we gain a greater understanding of the relationship between neuroaesthetics and the CATs, the framework of which adopts the “4E” approach to cognition: embodied, extended, embedded, and enactive ( King & Parada, 2020). Although the authors speak primarily in regard to art therapy, they proposed that research using MoBI with other CATs, such as dance/movement therapy could be beneficial in further understanding the underpinnings of mind-body connection as it relates to active art (or dance) making. The use of MoBI supports examination of the mind, body, behavior connection in a more natural setting of artistic practice; furthermore, the therapeutic processes and active factors of therapy have proven to be difficult to capture and can be explored with MoBI. “Importantly, the brain autonomously and continuously senses the body (i.e. interoception). MoBI, allowing the acquisition of brain/body physiological signals during a natural therapeutic encounter, opens the door for studying *interoception in the wild”* ( King & Parada, 2020, p. 8367). Furthermore, such research could bring greater awareness to the cognitive processes that inform artistic or aesthetic experiences as they relate to one’s sense of self, agency, and efficacy.

### Dance/movement therapy

Dance/movement therapy (DMT), rooted in nonverbal communication and dance as a healing factor, is an under-researched approach to increasing mind-body connectivity. Although not solely identified as a mind-body therapy, DMT does provide the opportunity to work on a holistic integration of the mind, body, and spirit, which could support mind-body integration and interoceptive awareness for participants. Dance/movement therapy is defined by the American Dance Therapy Association (2016) as “the psychotherapeutic use of movement to promote emotional, social, cognitive, and physical integration of the individual for the purpose of improving health and well-being” ( https://www.adta.org). Researchers have examined the effects of DMT on health outcomes with various populations resulting in positive changes towards health.

### Dance/movement therapy and health effects

Dance/movement therapy has been implemented as an intervention for physical health ( Conceição & Neto, 2016; Lopez-Nieves & Jakobsche, 2022; Neto *et al.,* 2014), mental health ( Karkou *et al.,* 2019; Koch *et al.,* 2019), autism ( Takahashi *et al.,* 2019), and trauma ( Levine & Land, 2016; Tomaszweski *et al.,* 2023) amongst other considerations. Systematic reviews and meta-analyses reviewed the effects of dance/movement therapy on blood pressure ( *K* = 4) ( Conceição & Neto, 2016) and chronic heart failure ( *K* = 2) ( Neto *et al.,* 2014). Results from both studies indicated an improvement in exercise capacity. Further results of Conceição and Neto (2016) study showed a significant reduction in both systolic and diastolic blood pressure as compared to the controls. Neto *et al.* (2014) conducted a review ( *K* = 17) on the biomoloecular effects of dance and DMT oh health and wellness. The included studies implementing DMT interventions resulted in an increase in serum nitric oxide and serotonin levels, and a decrease in dopamine and morning cortisol levels. These results could lead to improved cardiovascular health and decreased depression and stress. Outcomes measures showed an improvement in mental health scores following a DMT intervention.

Koch *et al.* (2019) conducted a meta-analysis ( *K* = 41; *N* = 2,374), which indicated that DMT has proven to be an effective psychosocial treatment option for a number of psychological and health related outcomes. The DMT studies in this meta-analysis yielded results that showed significant improvement quality of life, affect, cognitive skills, and interpersonal skills providing consistent medium effect sizes. Karkou *et al.’s* (2019) systematic review and meta-synthesis ( *K* = 8; *N* = 351) provided results of a decrease in depression for participants up to 3 months following participation in a DMT intervention.

Dance/movement therapy interventions have gone beyond physical and mental health improvements as seen in a systematic reviews conducted by Takahashi *et al.* in 2019 ( *K* = 7) and Tomaszweski *et al.* in 2023 ( *K* = 15) and the 2016 meta-synthesis by Levine and Land ( *K* = 9). Dance/movement therapy interventions showed promising results in improving social skills for autistic participants ( Takahashi *et al.,* 2019), particularly when attuning to participants using mirroring techniques. When considering DMT as an intervention for psychological trauma, participants reported an increase in mind-body connection, range of motion signifying increased agency ( Levine & Land, 2016), increased relaxation, decreased stress, improvements in bodily sensations and perceptions, increased emotional identification, expression, and externalization ( Tomaszweski *et al.,* 2023), and healthy intra- and interpersonal relationships ( Levine & Land, 2016; Tomaszweski *et al.,* 2023).

### Dance/movement therapy and equity

Equity and justice work has been a prominent and prioritized topic of interest amongst dance/movement therapists and researchers within the current literature. Dance/movement therapists have been developing trauma informed, justice driven theoretical models for DMT ( Jorden, 2022), exploring the role of power dynamics within an embodied activism approach ( Cantrick *et al.,* 2018), and considering and exploring critical consciousness in DMT pedagogy ( Kawano & Chang, 2019). There has been an increasing focus on cultural bias in movement repertoire and movement assessment tools as used in DMT. A focus on Black clients, Black experiences, Black historical context, and Black and Africanist Aesthetics in movement have been at the forefront of recent work. Nichols (2019) focused on addressing cultural and movement bias that may affect the understanding of the movement repertoire of Black participants in DMT. Whyte and Barclay (2019) discussed the incorporation and normativity of hip movements in movements derived in the African Diaspora. They compared to this to the oversexualized interpretation of the use of the hips in non-white derived movements and many movement assessment tools utilized by dance/movement therapists ( Whyte & Barclay, 2019).

Campbell (2019) considered the specific factors that affect African Americans including trauma, oppression, racism, transgenerational trauma, and slavery. With these phenomena in mind, Campbell (2019) developed a DMT-informed program for African American adolescents. The program was inspired by Dr. Joy DeGruy’s theory on Post Traumatic Stress Syndrome and incorporates dance as a way to address specific needs of African Americans to support healing, particularly as it relates to multigenerational trauma.

Kerney *et al.* (2023) named the significance of dance as a modality for healing for individuals of African descent, particularly Black womxn. The authors (2023) noted:

The letter ‘x’ in Black womxn centers sexual and gender minority (SGM) individuals like queer, transgender and nonbinary (TGNB), gender expansive folx, femmes, and all SGM people whose lived experience aligns with the sociocultureal experience of Black womanhood/womxnhood regardless of gender identity. (p. 2)

This content analysis ( *K* = 96) aimed to explore the presence of Black womxn in DMT research, noting their underrepresentation in the literature. Of the included studies, only 7.3% of them included Black womxn as participants. It is recommended that DMT can be a useful intervention for Black womxn and therefore, more research with this population is warranted.

### Dance/movement therapy and schizophrenia

The effects of DMT on symptom management for people with schizophrenia has shown promising results for people with both acute ( Biondo *et al*., 2021) and chronic ( Bryl *et al*., 2020) presentations of the diagnosis. Recent research has shown DMT to be effective in reducing positive ( Biondo *et al*., 2021) and negative symptoms of schizophrenia ( Biondo *et al*., 2021; Bryl *et al*., 2020; Gökcen *et al.*, 2020; Savill *et al*., 2017). Of particular interest for this review are the following additional results yielded from the research studies: increased social connectivity ( Biondo *et al*., 2021; Bryl *et al*., 2020; Gökcen *et al.*, 2020); mind-body awareness, and self-awareness ( Biondo *et al*., 2021; Bryl *et al*., 2020).

In our previous work, we examined the effects of a single-session DMT intervention for people with schizophrenia who were currently in an inpatient psychiatric facility due to symptom exacerbation ( Biondo *et al*., 2021). This mixed methods feasibility study ( *N* = 28) showed promising results in the diminishment of psychological discomfort and positive and negative symptoms of schizophrenia, as measured by the Brief Psychiatric Rating Scale, for participants randomized to a DMT intervention versus those in a treatment as usual control group. The qualitative findings substantiated the quantitative findings and provided complementary data in which participants reported increased interpersonal skills and feeling a sense of belonging. This is particularly pertinent based on the aforementioned stigmatization people with schizophrenia are faced with, contributing to disrupted interpersonal relationships. Both Biondo *et al*. (2021) and Bryl *et al*. (2020) reported an increase in self-awareness inclusive of mind-body awareness in these studies.

Bryl *et al*. (2020) conducted a mixed methods randomized controlled trial ( *N* = 31) providing 20 group DMT sessions over 10 weeks. Although the quantitative results of this study did not show significant findings, the qualitative data provided rich accounts of participant experiences in the DMT sessions. Participants articulated a greater awareness of self-integration including an increase in mind-body connectivity, and an increase in awareness of body boundaries ( Bryl *et al*., 2020). A similar theme was noted in our 2021 research, as participants shared feelings of increased self-awareness, particularly as it related to a positive change in their symptomatology. Increased self-awareness often led to reports of an increase in self-confidence and self-efficacy ( Biondo *et al*., 2021; Bryl *et al*., 2020).

Following their respective study interventions, participants expressed an increase in motivation to engage in more activities ( Bryl *et al*., 2020) and further treatment ( Biondo *et al*., 2021). A desire to continue activation may have been inspired by increased insight around felt changes from the sessions ( Biondo *et al*., 2021), and a connection participants made between physical and mental health ( Bryl *et al*., 2020).

In a 2020 systematic review, Millman and colleagues ( *K* = 15, *k* = 5, schizophrenia) examined the strengths and limitations of DMT as a treatment approach to various psychiatric populations. Particularly in reference to the studies with individuals with schizophrenia, it was determined that DMT may be an effective treatment option for reduction of positive and negative symptoms of schizophrenia and negative expressions of anger, as well as provide improved psychosocial functioning. Although this systematic review did not include the most recent research ( Biondo *et al*., 2021; Bryl *et al*., 2020; Gökcen *et al.*, 2020), it does conclude that DMT can be situated within a neurocognitive framework for future research on symptom diminishment of psychiatric disorders (Millmann *et al.*, 2020).

### Contributing factors

Tenets of the embodied aesthetics framework ( Koch, 2017), affective and embodied neurobiology ( Homann, 2010, 2020), and the embodied–enactive–interactive brain ( Vaisvaser, 2021) underlie the foundations of DMT that may support wellness for people with schizophrenia. For many years of practice in the creative arts therapies (CATs)—dance/movement, music, and art therapy—there have been notable gaps in theoretical frameworks that explained the active factors that contributed to wellness ( Koch, 2017). In response to this, Koch (2017) focused her research on developing the embodied aesthetics framework in which “the body is seen as a living organism (organismic metaphor), a unity with multiple interfaces to the environment and other persons, constituting emergent superordinate units beyond the person” (p. 86).

### Embodied aesthetics

The embodied aesthetics framework situates and overlaps the following components: bodily consciousness; environmental interactions, and an active body as a center for knowledge. Rooted in this theory, Koch (2017) identified five groupings of identified active factors that contribute to wellness: (1) hedonism; (2) aesthetics; (3) (nonverbal) meaning making; (4) enactive transitional support; and (5) generativity. Within each of these active factors, further detail is provided to encapsulate the contributors to wellness more fully.

Improvisation and playfulness are highlighted as significant components of hedonism, lending to creativity, strength, self-efficacy, and interpersonal connectivity. The role of aesthetics is not only to produce beauty, but also to feel confident that one creates beauty. Authenticity of movement provides self-efficacy for the mover, while allowing someone to truly be seen and fostering mind-body connection. Such nonverbal communication is a premise of DMT. In Koch’s (2017) framework, this can be sectioned into cognitive, affective, and transpersonal symbolizing. In the former, participants of DMT are able to cognitively organize and integrate symbols that emerged from the dance. In affective symbolizing, the dance becomes a vehicle for processing and expressing emotions. In the latter, the dance becomes a spiritual bridge connecting the mover to the understanding of universal cohesion and a place for ritual to form. Through these processes, the movement creates and maintains containment for the participant(s) providing safety in expression and a trajectory towards wellness. With this enactive transitional support, movers can develop agency, re-establish self-safety, and encourage activation. The final category of generativity serves the mover in being a creator, which fosters self-efficacy, agency, continued activation, and resilience ( Koch, 2017). Together, the components of the embodied aesthetics provide support for increased mind-body connection and physical and mental wellness.

### Embodied neurobiology

The experiential nature of DMT lends towards an embodied neurological approach that connects the mind and body through movement experiences and nonverbal psychological processing. The collaborative creation of in-the-moment intervention choices made between participant and therapist allow agency and therapeutic rapport to be at the forefront of DMT sessions. Homann (2020) theorized five components to the embodied neurological approach to DMT: (1) polyvagal and biochemical regulation; (2) interoception; (3) empathy and attunement; (4) memory and affective systems and (5) brain lateralization. Priority is placed on the mind-body connection as a contributing factor to accessing one’s emotional intelligence and defining a healthy sense of self. Furthermore, Homann (2020) found:

DMT’s unique emphasis on experiential engagement of the body has significant therapeutic implications, engaging the mind from the inside out. Movement engages deep systems of biochemical regulation, facilitates arousal and rest, and stimulates the core of self–perception at the neurological intersections of emotional, sensory, and cognitive processes. (p. 298)

Dance/movement therapy informed by Polyvagal theory promotes the simultaneity of activation and rest and is often fostered by safe social engagement. Such safety is developed through attunement and can be experienced through touch and gaze ( Homann, 2010). Gray (2017) referred to the “safety–trust–relationship continuum” in her framework for Polyvagal–informed DMT as a foundational component to shifting physiological, and thus psychological states in the therapeutic process (p. 44). Early ruptures in attachment can manifest though affective, cognitive, and physical reactions. With this in mind, it is imperative that the repair processes access each of those manifestations, which is possible through DMT ( Gray, 2017; Homann, 2020). Through the DMT process, participants develop an increase in body awareness while cultivating feelings of relaxation, self-regulation, and agency. Moreover, as individuals receiving treatment create a space of relative safety, they are provided with greater access to a healthy relationship with their body. The therapeutic process of dance and movement within a DMT session can improve interoception, thus providing functional access to body and neurological activation, resulting in greater understanding of physical and emotional needs ( Homann, 2020).

This process of developing greater awareness of self can then transfer to fostering healthier and more productive interpersonal relationships. Attachment is developed through empathic connections, and nonverbal communication is a pathway through which mirror neurons are activated ( Homann, 2010). Kinesthetic attunement or embodied empathy is experienced when a dance/movement therapist and client engage in mirroring one another’s movements, a common technique of DMT. Imparting a sense of being seen in this way for people with schizophrenia validates their authentic selves and instills a sense of pride and feelings of belonging ( Biondo *et al*., 2021).

A relationship substantiated by attunement, empathy, and authenticity allows for depth of processing through nonverbal experiences including intersubjectivity and integration of implicit and explicit memory. The nonverbal attunement and neuronal matching experienced collectively by dance/movement therapists and clients creates safety and the capacity for sharing unconscious material through intersubjective processes ( Homann, 2010). This intersubjective experience “activates the mirror neuron system, and, through consequential neuronal, hormonal, and chemical cascades connecting the limbic system, the autonomous nervous system and the right hemisphere’s orbitofrontal cortex, facilitates the experience of being with another, in a conscious manner” ( Homann, 2010, p. 90). This integrative process happens on emotional, sensorial, and cognitive levels.

Integration expands to that of memory as implicit memory can be stored in the body and elicited through the embodied processes of DMT and become explicit. This process not only allows recollection of memory, but it also provides an environment for emotional processing and re-narration to create a place of safety for deeper exploration and healing ( Homann, 2020). Furthermore, integration goes beyond that of implicit and explicit memory and extends to lateralization of the brain. Movements, particularly those which crosses the midline and engage the whole body, support an integration of brain hemispheres providing lateralization, whole brain connectivity, and thus integration of self ( Homann, 2020).

### Embodied–enactive–interactive Brain

The embodied–enactive–interactive brain framework is an integrative approach of the CATs with fundamentals of neuroscience that takes similar approaches to the two aforementioned frameworks. Vaisvaser (2021) theorized that the mind, body, and environment intersect which creates a space for integration and thus wellness. Furthermore, exploration of brain functions as they relate to therapeutic factors are presented in five sections: (1) embodiment through a neuroscientific lens; (2) “predictive nature of the mind and the formation and reformation of internal models”; (3) “predictive processing mechanisms in the context of psychic apparatus”; (4) “developmental and therapeutic implications of the brain’s predictive mechanisms”; and (5) “the relational account of neural functioning and the underpinnings of empathy” ( Vaisvaser, 2021, p. 2). Underlying the embodied–enactive–interactive brain is the premise that mind, body, and interpersonal relationships are the root of healing processes for which the CATs are a vehicle.

The concept of an embodied brain suggests that the body—and particularly interoceptive and proprioceptive awareness—plays an active role in cognitive processes. Sensory experiences of the body are not only a receptive vessel for informational absorption, but also activators for neural indicators of sensory, motor, emotional, and linguistic information ( Vaisvaser, 2021). The environmental component of this framework aligns with social engagement in Polyvagal theory, placing value on the relational component of cognitive and emotional processes. With that, the intersectionality of our mind–body–relational being provides a platform for perception of self and other through creative processing. The predictive brain has a complex and multisensory relationship with the body and environment. Brain prediction, or the “embodied brain,” in which the brain reflects on past experiences in order to determine probable outcomes, incorporates interoceptive knowledge to predict movement, intention, and emotion, and to further agency, self-efficacy, and self-awareness ( Vaisvaser, 2021).

Vaisvaser (2021) theorized that an embodied or predictive brain encourages and supports advancement or progress within the therapeutic realm. Therefore, individuals engaged in the CATs connect with interoceptive and exteroceptive sensory information, which, through processing, increases emotional awareness and expression. Per Vaisvaser (2021) this then contributes to activation, disrupting the potential for returning to a homeostatic place of stagnancy. The CATs provide a structure for positive disruption of that which is familiar and lead to the creation of new opportunities. Dance/movement therapy can thus be an opportunity for emotional expressivity, externalization, and therapeutic meaning–making ( Biondo *et al.*, 2021; Vaisvaser, 2021). Furthermore, re–narration of past traumatic events can provide an opportunity for integration and insight. This forward movement is further cultivated by a safe environment and therapeutic rapport ( Vaisvaser, 2021).

Vaisvaser’s (2021) review suggests that these introspective and intrapsychic experiences are deepened through relational encounters both overt and intersubjective. Neurologically, our brains are wired to seek out social engagement. Verbal and nonverbal interactions are instrumental in healthy and adaptive brain functioning though activation of mirror neurons and neuronal synchronization ( Vaisvaser, 2021). These processes aide in an increase in intra- and interpersonal emotional awareness, as well as the release of oxytocin, supportive of adaptive attunement. Empathy, kinesthetic and otherwise, creates pathways for intersubjective processes and mentalization. “Importantly, mentalization during empathic engagement refers to the attribution of emotions, wishes, desires, and needs … suggesting that distinct neural networks are involved in self-knowing and knowing others…” ( Vaisvaser, 2021, p. 6–7). Moreover, shared moments of synchrony through kinesthetic empathy establish foundational tools for rapport building, co–regulation, healthy attachment, and resilience, particularly when rooted in arts and artistic experiences. Vaisvaser (2021) suggested the field of neuroimaging to better understand the distinct processes of neural activity.

## Conclusions

Individuals with schizophrenia face disproportionately high barriers to health and wellness. Discrepancies in health care for this population begin with diagnostics ( Asonye *et al.,* 2020; Metzl & Roberts, 2014; Oud & Jong, 2017) and extend through the duration of the illness, affecting symptomatology, intra- and interpersonal skills, quality of life, self-efficacy, and overall prognosis ( Kohn *et al.*, 2022; Rössler, 2016; Vancampfort *et al.*, 2017). People of color, particularly Black men, have historically been most affected by diagnostic inequities and biases ( Alang, 2019; Asonye *et al.,* 2020; Harnett & Ressler, 2021; Metzl & Roberts, 2014; Misra *et al.,* 2022). Furthermore, people with schizophrenia face high rates of stigmatization, not only by the general population, but also by the health care providers by whom they are seeking services ( Ivanova, 2021; Kohn *et al.*, 2021; Rössler, 2016). These stigmatizing behaviors, coupled with communication differences that people with schizophrenia may experience, deter people with schizophrenia from seeking and receiving quality health care ( Kohn *et al.*, 2022). Moreover, their propensity towards a dissociative relationship with their bodies and additional bodily dysregulations places this population at a proportionately higher risk for medical illness ( Moore *et al.*, 2015; Oud & Jong, 2017; Tan *et al.*, 2021) resulting in a significantly shorter life span than the general population ( Kohn *et al.*, 2022; Swildens *et al.*, 2016; Tan *et al.*, 2021).

The cyclical nature of physical and mental symptomatology triggering one another indicates that improved mind-body awareness could be a supportive option for people with schizophrenia ( Costantini *et al.*, 2020). Recent research has shown that this population is particularly vulnerable to experience diminished understanding of their mind-body connection ( Kohn *et al.*, 2022; Yao & Thakkar, 2022) and a decrease in interoceptive knowledge ( Ardizzi *et al*., 2016; Torregrossa *et al.*, 2022; Yao & Thakkar, 2022). This limits the ability to process internal and bodily signals and information that could provide insight around physical health needs. Much of the body–mind connection research focuses on externalization and could benefit from an inside-out perspective initiating interventions from the body and making cognitive connections after participants have a bodily, felt experience. This process would support both physical and mental aspects of care benefitting both general and mental health care, both of which are needed for people with schizophrenia.

Dance/movement therapy is a treatment option that addresses physical ( Conceição & Neto, 2016; Lopez-Nieves & Jakobsche, 2022; Neto *et al.,* 2014), mental ( Biondo *et al.,* 2021; Bryl *et al*., 2020; Karkou *et al.,* 2019; Kerney *et al.*, 2023; Koch *et al.,* 2019; Levine & Land, 2016; Milman *et al.,* 2021; Tomaszweski *et al.,* 2023), and social aspects of participant wellness ( Biondo *et al.,* 2021; Bryl & Biondo, 2022; Takahashi *et al.,* 2019). More recently, DMT frameworks have been explored and expanded to be inclusive of embodied, neurophysiological theories. Of note is the inclusion of neurophysiological theories which incorporate concepts of attunement and self-regulation both of which play a significant role in DMT ( Homann, 2010, 2020). Due to the direct relationship between the vagus nerve and interoception ( Homann, 2020), DMT would be an apparent intervention choice. The onto-epistemology of DMT tells us that the body, dance, and movement are tools that inform the needs of the holistic self. As an active and embodied intervention, the research discussed demonstrates that DMT has the capacity to address multi-layered needs of people with schizophrenia including raising interoceptive awareness. As research has shown, DMT for people with schizophrenia can help participants become activated through movement ( Bryl *et al*., 2020), improve interpersonal relationships, process and express emotions, improve self-efficacy, and increase mind-body connection ( Biondo *et al*., 2021; Bryl *et al*., 2020). Dance/movement therapy is an active intervention in which people experiencing all levels of acuity can participate ( Biondo *et al.*, 2021). Moreover, the nonverbal relationships possible through DMT can allow for increased joining and attuning with those who may have communication differences. These components of invitation for people with schizophrenia truly meet each participant where they are in their wellness process and destigmatize the diagnosis, allowing an authentic development of healthy ego, self-efficacy, and relationship ( Biondo *et al.*, 2021; Bryl & Biondo, 2022).

This review seeks to highlight the disparities people with schizophrenia face regarding lack of accessible ( Swildens *et al.*, 2016) and quality health care ( Moore *et al.*, 2015; Sølvhøj *et al.*, 2021), multifaceted forms of stigmatization they encounter ( Ivanova, 2021; Kohn *et al.*, 2022; Sølvhøj *et al.*, 2021), and specific barriers they face regarding the bodily disruptions and dysregulations associated with their symptomatology ( Biondo *et al.*, 2021; Costantini *et al.*, 2020; Torregrossa *et al.*, 2022). Dance/movement therapy is an intervention that can provide individuals diagnosed with schizophrenia an opportunity to participate in an embodied psychosocial treatment intervention that addresses these precise body-based needs, while simultaneously providing physical activation, emotional processing, mind-body connection, interpersonal relationships, and self-regulation. These are components that not only support increased personal and interpersonal wellness, but also provide self-efficacy to encourage physical and mental health care adherence, and embodied treatment experiences placing mind-body wellness at the forefront of care ( Biondo *et al.*, 2021). A treatment protocol inclusive of DMT can provide a foundation for the rehumanization process that people with schizophrenia certainly deserve.

## Data Availability

No data are associated with this article.
