# Supplementary material for: Dance/movement therapy as a holistic approach to diminish health discrepancies and promote wellness for people with schizophrenia: a review of the literature
Source: F1000Res. 2023 Aug 1;12:33. Originally published 2023 Jan 9. [Version 2] doi: 10.12688/f1000research.127377.2 (PMC10429376; doi:10.12688/f1000research.127377.2)
Supplement: Peer review history

# Peer review: Reviewer response for version 2

**DOI:** 10.5256/f1000research.153811.r192854

**Published:** 2023-08-15

**Authors:** Robyn Cruz

**Affiliations:** 1 Division of Expressive Therapies, Lesley University, Cambridge, MA, USA

The author has addressed concerns related to organization.

Is the review written in accessible language?

Yes

Are all factual statements correct and adequately supported by citations?

Partly

Are the conclusions drawn appropriate in the context of the current research literature?

Yes

Is the topic of the review discussed comprehensively in the context of the current literature?

Yes

Reviewer Expertise:

Dance/movement therapy, movement disorders, research methods

I confirm that I have read this submission and believe that I have an appropriate level of expertise to confirm that it is of an acceptable scientific standard.

# Peer review: Reviewer response for version 2

**DOI:** 10.5256/f1000research.153811.r192853

**Published:** 2023-08-01

**Authors:** Hod Orkibi

**Affiliations:** 1 Faculty of Social Welfare and Health Sciences, University of Haifa, Haifa, Haifa District, Israel

The paper was revised according to the suggestions of the reviewers.

Is the review written in accessible language?

Yes

Are all factual statements correct and adequately supported by citations?

Partly

Are the conclusions drawn appropriate in the context of the current research literature?

Yes

Is the topic of the review discussed comprehensively in the context of the current literature?

Yes

Reviewer Expertise:

Psychodrama and drama therapy, arts therapies, community mental health, process and outcome research, systematic reviews.

I confirm that I have read this submission and believe that I have an appropriate level of expertise to confirm that it is of an acceptable scientific standard.

# Peer review: Reviewer response for version 1

**DOI:** 10.5256/f1000research.139878.r169980

**Published:** 2023-04-28

**Authors:** Hod Orkibi

**Affiliations:** 1 Faculty of Social Welfare and Health Sciences, University of Haifa, Haifa, Haifa District, Israel

Overall, the article focuses on a timely issue of offering DMT to clients with schizophrenia, which is generally an under-investigated field. 

 In the Discussion, address clearly the unique contribution of DMT to this population, and how modality-specific change factors are reasoned to lead to outcome. In other words, based on the literature reviewed, it will be meaningful if you specify your own  theory of change with respect to DMT for this population. 

 Also, the polyvagal theory has been criticized in the literature, and this has to be mentioned. 

 The abbreviation CATs should appear in the first time the term "creative arts therapies" - it appears under: "Contributing factors". This should be followed by CATs. 

 The Conclusion section can be more concise in highlighting the take home message of this review to strengthen the impact of the paper.

Is the review written in accessible language?

Yes

Are all factual statements correct and adequately supported by citations?

Partly

Are the conclusions drawn appropriate in the context of the current research literature?

Yes

Is the topic of the review discussed comprehensively in the context of the current literature?

Yes

Reviewer Expertise:

Psychodrama and drama therapy, arts therapies, community mental health, process and outcome research, systematic reviews.

I confirm that I have read this submission and believe that I have an appropriate level of expertise to confirm that it is of an acceptable scientific standard, however I have significant reservations, as outlined above.

# Peer review: Reviewer response for version 1

**DOI:** 10.5256/f1000research.139878.r160297

**Published:** 2023-01-31

**Authors:** Robyn Cruz

**Affiliations:** 1 Division of Expressive Therapies, Lesley University, Cambridge, MA, USA

The scope of this paper is impressive and the arguments for providing care for individuals with schizophrenia that is aligned with care for the whole person continues to be a worthy topic for research and practice. The decades-long focus of pharmacotherapy as the primary treatment for schizophrenia continues to show progress, but the need for other treatment modalities such as dance/movement therapy (DMT) to address social and psychological components of the illness such as trauma are still required. This review will combine comments with a focus on strengthening the content to help achieve the worthy goals of the paper.

 Introduction – I am not completely clear about the meaning of the sentence “Through a strengths- and body-based approach, dance/movement therapy (DMT) surpasses traditional verbal communication, establishing inclusion for many forms of preferred communication styles (Biondo et al., 2021).” especially for readers who may not have an understanding of DMT. I think here in the introduction – an introduction to the field of DMT is warranted. The section that is on page 5 (of the pdf version of the article) could be moved to the introduction and I believe it would be very useful in tying together other sections of the paper for the reader new to DMT. The sections on body symptomatology, interoception, and mind-body treatments could be helped by setting the context early on with what DMT is and the fact that it has been used for decades with seriously mentally ill patients as well as others.

 Discussion – I feel that there needs to be more research cited, and more diversity of sources for the important discussion of mental health disparities. Currently there is sole dependence on Metzl & Roberts (2014). In addition the mention of the changes in rates of diagnosis over time fails to mention the important fact that diagnostic criteria have changed quite drastically over time as well. To cite myself  (Cruz, 1995, p. 38), “Yet a narrowing of diagnostic criteria for schizophrenia has taken place during the last 50 years, changing the determination of who is diagnosed schizophrenic. The *DSM-III *(1980) represented the sharpest break with previous editions in the definition and diagnostic criteria for schizophrenia (Andreasen & Flaum, 1991). Many forms of the disorder were removed and included in other categories, most notably psychosis not elsewhere classified, affective disorders, and related personality disorders. In spite of this narrowing, research on movement disorders has not regularly included the removed disorders.”

 In 1952 DMS I was published and the criteria for schizophrenia as noted above included other disorders that later were winnowed out. DSM II was published in 1968 and DSM III in 1980 – in spite of these changes, it would be good to note if disparities in diagnosis by race continued across the changing symptom criteria.

 Also the discussion on stigmatization – other sources should be cited as there is dependence on Rossler (2016). It could be helpful also to make a connection between the discussion on stigmatization and stigmatizing behaviors – it is possible combining the paragraph that begins “Stigmatization also affects people with schizophrenia on personal and social levels.” with the section located under the heading “physical health disparities” could be helpful for the reader.

 Think about the sections in “Mental Health Disparities” and “Physical Health Disparities” as setting up the reader for better understanding of why DMT has the potential to promote wellness for this group. I think it is necessary to distinguish better between “Body symptomatology” and “Bodily disturbances…” as what is described under body symptomatology are abnormal involuntary movements which can be naturally occurring or side effects of pharmacology but are related to dysfunction of neurotransmitters of the extrapyramidal system (Cruz, 1995; 2009). I also think that the text in the “Bodily disturbances section could more meaningfully be combined with the “Interoception” section of the paper – they cross-over in ways that might be confusing to the reader not familiar with these terms.

 I am curious what the real purpose of the  “Mind-body treatments” section is and if the author thinks of DMT as a mind-body treatment? If so this needs to be clarified for the reader so that the DMT research section (which is brief and does not include any meta-analyses that have been conducted) is clear and then clearly separated from the theoretical sections that follow. These theoretical areas “embodied aesthetics framework and embodied neurology need better framing as theoretical frameworks. How those frameworks might directly impact work with schizophrenic patients needs more obvious connection for the reader.

 I am also curious about the inclusion of biofeedback which is a bonafide technique and neurofeedback with seems to be theoretical – what could be learned or achieved with it? They don’t seem to “fit” very well with this paper.

 Conclusion – may serve as a good guide for the reorganizing that I’ve suggested in this review. It is comprehensive of the major points in a very clear manner that serves the paper well.

Is the review written in accessible language?

Yes

Are all factual statements correct and adequately supported by citations?

Partly

Are the conclusions drawn appropriate in the context of the current research literature?

Yes

Is the topic of the review discussed comprehensively in the context of the current literature?

Yes

Reviewer Expertise:

Dance/movement therapy, movement disorders, research methods

I confirm that I have read this submission and believe that I have an appropriate level of expertise to confirm that it is of an acceptable scientific standard, however I have significant reservations, as outlined above.

## References

[1] : An empirical investigation of the Movement Psychodiagnostic Inventory. *The University of Arizona, Tucson* .1995; Reference source

[2] : Validity of the Movement Psychodiagnostic Inventory: A Pilot Study. *American Journal of Dance Therapy* .2009;31(2) : 10.1007/s10465-009-9072-4 122-135 10.1007/s10465-009-9072-4
